# Contribution of Biofertilizers to Pulse Crops: From Single-Strain Inoculants to New Technologies Based on Microbiomes Strategies

**DOI:** 10.3390/plants12040954

**Published:** 2023-02-20

**Authors:** Gustavo Ribeiro Xavier, Ederson de Conceição Jesus, Anelise Dias, Marcia Reed Rodrigues Coelho, Yulimar Castro Molina, Norma Gouvêa Rumjanek

**Affiliations:** 1 Embrapa Agrobiologia, Rodovia BR-465, Km 7, Seropédica 23897-970, RJ, Brazil; 2 Departamento de Fitotecnia, Instituto de Agronomia, Universidade Federal Rural do Rio de Janeiro, UFRRJ, Rodovia BR-465, Km 7, Seropédica 23890-000, RJ, Brazil; 3 Programa de Pós-graduação em Microbiologia Agrícola, Universidade Federal de Lavras, UFLA, Trevo Rotatório Professor Edmir Sá Santos, Lavras 37203-202, MG, Brazil

**Keywords:** biological nitrogen fixation, PGPR, arbuscular mycorrhizal fungi, biofertilizers, sustainable production, plant–microbe interaction, nodule microbiome, rhizosphere

## Abstract

Pulses provide distinct health benefits due to their low fat content and high protein and fiber contents. Their grain production reaches approximately 93,210 × 10^3^ tons per year. Pulses benefit from the symbiosis with atmospheric N_2_-fixing bacteria, which increases productivity and reduces the need for N fertilizers, thus contributing to mitigation of environmental impact mitigation. Additionally, the root region harbors a rich microbial community with multiple traits related to plant growth promotion, such as nutrient increase and tolerance enhancement to abiotic or biotic stresses. We reviewed the eight most common pulses accounting for almost 90% of world production: common beans, chickpeas, peas, cowpeas, mung beans, lentils, broad beans, and pigeon peas. We focused on updated information considering both single-rhizobial inoculation and co-inoculation with plant growth-promoting rhizobacteria. We found approximately 80 microbial taxa with PGPR traits, mainly *Bacillus* sp., *B. subtilis*, *Pseudomonas* sp., *P. fluorescens*, and arbuscular mycorrhizal fungi, and that contributed to improve plant growth and yield under different conditions. In addition, new data on root, nodule, rhizosphere, and seed microbiomes point to strategies that can be used to design new generations of biofertilizers, highlighting the importance of microorganisms for productive pulse systems.

## 1. Introduction

The global challenge of feeding an 8 billion people population, which should reach 9.7 billion by 2050 [1], represents a planetary dilemma. Food supply depends on agricultural aptitude and soil management under the threat of climate change, germplasm production (animal, plants, and microorganisms), and geopolitical aspects among other aspects. Furthermore, food supply needs to be achieved in a complex environment where the increase in purchasing power has led to changes in the consumption pattern, favoring the consumption of animal protein, the inclusion of traditional and indigenous peoples and their cultures, and the commitment to world peace should be a permanent goal.

Pulses comprise a group of crops that are consumed as dry beans, rich in protein and fiber, which play an essential role in human nutrition, thus representing a valuable global food security resource [2]. Moreover, they perform critical biological functions such as anti-stress [3], anti-inflammatory [4], antioxidant, and liver protection [5]. Due to their related nutritional and healthy qualities, pulses are an alternative solution for reducing hidden hunger, frequently found in impoverished populations around the world who have a limited access to animal protein, vitamins, and minerals supplements [6]. Increasing the offer of plant-based protein, especially for the low-income populations, is a key to improving the quality of the diet at an affordable price. To further encourage vegetable consumption, actions based on plant-based proteins have gained prominence through technological innovation, aiming to approximate the flavors and textures of products developed from plants to animal-based food, expanding opportunities for consumption, and consequently stimulating production scaling and the adoption of new technologies to production systems [7,8]. In addition to being a significant contribution to eradication of hunger, pulses may improve human health and reduce soil degradation. Regarding these characteristics, they are an associated component towards the achievement of the 2030 Agenda for Sustainable Development and Sustainable Development Goals (SDGs) [9].

Among several vegetables that are classified as pulses, the temperate legumes chickpeas, peas, broad beans, and lentils, and the tropical legumes beans, cowpeas, mung beans, and pigeon peas are the subject of this review. The total pulse production from 2018 to 2020 accounted for approximately 93,210 × 10^3^ tons per year, while the eight pulses listed above represent approximately 88% of the total (Table 1) [10].

**Figure 1:**
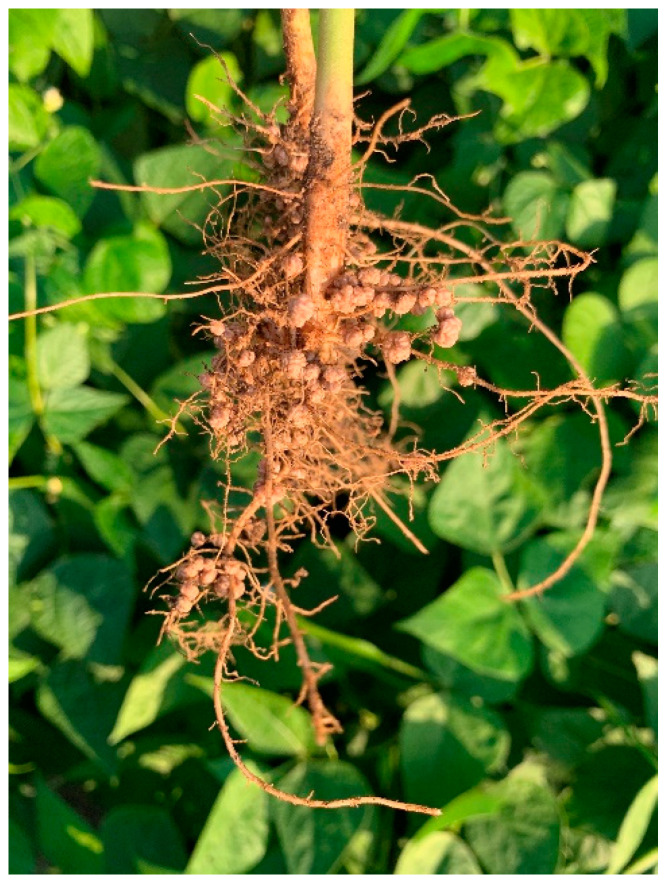
Nodulated root of *Phaseolus vulgaris*. Credits: Ederson da Conceição Jesus.

Most pulses are especially suitable for production in arid or subtropical areas since they grow in soils with low natural fertility and are capable of tolerating drought, salinity, and thermal stresses. In part, this ability to thrive in low-fertility soils is given by biological nitrogen fixation (BNF) carried out by rhizobia that inhabit the root nodules of these legumes (Figure 1), as well as the association with other plant BNF activity by microsymbionts inhabitant of pulse nodules-promoting rhizobacteria (PGPR). In this text, PGPR includes both bacteria and fungi living in roots, nodules, and the rhizosphere. The interest in the nodule microsymbionts, and other PGPR, has steadily increased in recent years. 

There is a wide literature related to PGPR activity and the associative models between host plant and microorganisms elaborated through anatomy, biochemistry, and physiology mechanisms [11,12,13,14,15,16,17,18,19,20,21,22]. The interaction between the host plant and the microsymbiont may produce symbioses with different levels of efficiency, so research has been intensely concerned with recognizing and selecting the best symbioses and associations to increase the percentage of fixed N. Pulses may also be responsive to PGPR aiming to increase yields and nutrients either under edaphic and climatic conditions recommended for each crop or under climatic stress conditions.

This work aims to review the most common strategies that have been carried out to optimize biological processes for the eight most produced pulses worldwide through a systematic analysis using the Web of Science database, mainly during the 2018–2023 period. The selected studies range from classic assays with isolated strains, through multiple functional inoculants, to knowledge acquired from microbiome approaches. The emphasis was placed on biological functions such as BNF, nutrient solubilizers, 1-aminocyclopropane-1-carboxylate (ACC) deaminase activity, and the production of indole-3-acetic acid (IAA), and siderophores, related to non-stressed or stressed environments (salinity, thermal, and drought). In this review, a close look has been placed on the current scenario of the microbial species that are being tested in different assays, with the aim of understanding the predominant groups, which show potential for the development of future technologies that could contribute to the increase in pulse productivity.

## 2. Microsymbionts Inhabitants of Pulse Nodules 

Microsymbionts are microbial endophytes that inhabit the interior of plant tissues without causing any damage to the host plant. A group of microsymbionts that inhabit legume nodules characterizes a symbiosis capable of fixing N^2^ from the air [18,19]. BNF activity is mediated by an intense exchange of molecular signals, including flavonoids and bacterial lipooligosacharides, leading to nodule formation.

### 2.1. Rhizobium Leguminosarum Symbiovar Viciae (Rlv) Strains: The Main Microsymbiont for Peas, Lentils, and Broad Beans

Peas, broad beans, and lentils are nodulated by a wide variety of soil *Rhizobium leguminosarum* symbiovar *viciae* (Rlv) strains, although other rhizobial species can also promote the formation of nodules in these crops [23,24]. According to host specificity, *R. leguminosarum* comprises two other symbiovars, *trifolii* and *phaseoli* [25]. Due to the large diversity of bacterial strains, Rlv is described as a “species complex” (Rlc), which includes 18 distinct genospecies and seven unique strains [26]. Five of the genospecies include type strains of *Rhizobium laguerreae*, *Rhizobium sophorae*, *Rhizobium ruizarguesonis*, *Rhizobium indicum*, and *R. leguminosarum* itself. More recently, the nodulation of pea by *Sphingomonas sediminicola* has been reported, describing the first example of nodulation of legumes with this genus [24].

The soil type and the variety of pea significantly influence the efficiency of native rhizobia, nodulation, shoot and root biomass production, and accumulation of shoot nitrogen [27]. Boivin et al. [23] claimed that competitive rhizobia are not necessarily the most efficient at fixing atmospheric nitrogen, which can often result in a crop yield reduction. They concluded that competitiveness was associated with specific regions of the *nod* locus without a relationship to variations in the core genome. Furthermore, Boivin et al. [23] co-inoculated a collection of 32 Rlv strains in three genotypes of each host species: peas, broad beans, and lentils. From the results, they estimated the Early Partner Choice (EPC) strain for the Fabeae tribe hosts, also known as tribe Vicieae. Most of the tested Rlv strains induced nodules in all the Fabeae hosts, but their behavior is distinct when they are co-inoculated. For that reason, when it comes to inoculation of Rlv strain, it is common to repeat the inoculation at every new planting to guarantee adequate crop yields [27,28].

Climate variability and farming management practices with a heavy reliance on fertilizers and herbicides can result in a reduction in soil carbon, fertility, and pH, and not rarely, can contribute to an increase in the incidence of plant disease. In legume production areas where rhizobial contribution to BNF is at risk, a solution was sought based on the selection of Rlv strains by superior symbiotic performance and saprophytic competence [29]. A large rhizobial collection obtained from acid soils was the source for fourteen strains with a superior BNF capacity, higher than 78% nitrogen derived from the atmosphere, and twenty-two strains that showed a high soil survival capacity into the next season [30]. From this collection, the authors performed a selection regarding both traits in comparison to commercial strains, which achieved only 3% of nodule colonization; they found an elite strain, WSM4643, characterized by its better survival ability, which may represent a potential inoculant for peas in dry soil agricultural areas. 

Mendoza-Suárez et al. [31] used reporter plasmids to differentiate rhizobial nodulation competitiveness from BNF effectiveness in individual nodules by using green fluorescent protein (GFP) and barcode strain identification (plasmid ID). After monitoring 84 Rlv strains, a super-competitive and highly efficient strain for peas was found. Furthermore, the method has shown that nodules colonized by multiple strains were quite common, including one nodule with six different strains. Standardization of the technique may provide a quick way to characterize nodule-forming rhizobia from agricultural systems, helping to improve the benefits of biofertilizers.

Phylogenetic characterization of a bacterial collection isolated from broad beans and lentils cultivated in ten different sites in Ethiopia identified nine genospecies of Rlv, suggesting the presence of a diverse rhizobial population associated with the Viciae tribe [32]. The genospecies distribution was mainly due to environmental variables rather than to the host legume species. Alternatively, in China, broad bean rhizobia belong to different *Rhizobium* species, such as *R. anhuiense*, *R. fabae*, *R. vallis*, *R. sophorae*, *Agrobacterium radiobacter*, and four strains related to *Rhizobium* and *Agrobacterium* [33]. Analysis of the plant growth promotion and symbiotic genes (*nifH* and *nodC*, respectively) revealed the presence of a locally adapted diversity of broad bean rhizobia.

Indigenous rhizobial population isolated from broad bean nodules from different regions of Tunisia showed tolerance to salinity and pH variation, in addition to BNF and phosphate (P) solubilization activities [34]. Another rhizobial collection from broad beans isolated from cultivated lands in Egypt also consisted of salinity stress-tolerant strains and P solubilizers [35]. Some strains, even under salt stress, showed growth-promoting characteristics and may have the potential to be used as a biofertilizer for areas presenting abiotic stresses.

A two-year field experiment with broad beans was conducted in Tunisia, where crop cultivation is declining due to strongly acid soil (pH 5.1) and a low available P content [36]. Inoculation with an indigenous strain showed the highest yield, 3137 kg ha^−1^, compared to the uninoculated control, representing an increase of almost 40%. Another two-year multilocation experiment using six indigenous rhizobial strains as inoculants and three varieties of faba beans resulted in an increase in nodulation and shoot and root dry weight [37]. The results were greatly influenced by the interaction between the three factors: location, strain, and variety [37]. The results obtained by Lishan et al. [38] showed that at 50 cm spacing between rows, the inoculation of broad beans increased grain yield more than twice compared to the control without inoculation, achieving 2540 kg ha^−1^. 

In Morocco, nodules from lentil accessions were a source of fourteen *Rhizobium* strains [39]. Phylogenetic analysis grouped them into three rhizobial species, *R. laguerreae*, *R. leguminosarum*, and *Mesorhizobium huakuii*, represented by just one strain. One of the varieties showed the best association with ten strains, leading the authors to conclude that the lentil genotypes largely ruled both rhizobial genetic diversity and symbiotic efficiency. 

In semi-arid areas, lentil seeds were inoculated with rhizobial strains obtained from nodules of two cultivars of peas and lentils growing in sub-humid and semi-arid regions [40]. The results showed that lentil inoculation with the strains from sub-humid areas were more effective in promoting plant development under conditions of environmental stress. 

In a field experiment carried out in Saskatoon, Canada, for two consecutive years, lentil inoculation with a commercial granular rhizobial formulation, Nodulator (strain P2) showed results similar to N fertilizer application (50 kg ha^−1^), and for the uninoculated control, around a 20% increase in yield was observed [41]. 

### 2.2. Rhizobium spp. Strains: The Main Microsymbionts for Common Beans

The common bean nodulates preferentially with the genus *Rhizobium,* in contrast to what has been observed for other species of *Phaseolus* that nodulate preferentially with *Bradyrhizobium* [42]. This observation has been made mainly with culture-dependent methods. More recently, Bender et al. [43], using metabarcoding, observed that *Rhizobium* dominated in the nodules of uninoculated common beans in the field, supporting the preference of the crop for this genus.

The common bean is known for its promiscuity, which according to Shamseldin and Velázquez [42] is attributed to its ability to nodulate with different symbiovars, including *phaseoli* and *tropici*, *gallicum*, *giardinii*, and *mediterranense*. Two major symbiovars, *phaseoli* and *tropici*, were first found in the Americas. *R. etli*, which, together with *R. leguminosarum* and *R. phaseoli*, constitutes a major phylogenetic clade within the genus, is the main symbiont in diversification centers [44]. *Rhizobium tropici* and related species are adapted to acid soils and are found in Brazil, Colombia, Chile, and Argentina. The other symbiovars were first found in Europe, Africa, and Asia [42]. Although dominant under some conditions, these symbiovars are shared between different locations, indicating the exchange of rhizobial diversity and the exchange of genetic material through horizontal gene transfer. 

In addition to nodulating with different *Rhizobium* symbiovars, common bean nodulates with a wide range of rhizobial genera and species, including alpha- and beta-rhizobia. Among these are *Ensifer* spp., *Pararhizobium* spp., *Bradyrhizobium* spp., *Cupriavidus* spp., and *Paraburkholderia* spp. [42,45,46]. However, the interaction with these species occurs at different effectiveness levels. An effective symbiosis is usually established with *Rhizobium*, *Ensifer*, and *Pararhizobium* [42]. *Bradyrhizobium* spp. can induce nodulation, but the nodules are usually not colonized effectively, with strains remaining in the intercellular space and not entering cells to form an effective symbiosis [47]. However, at least one effective symbiosis has already been reported [48]. Beta-rhizobia, such as *Paraburkholderia* and *Cupriavidus*, can also nodulate the common bean, usually with a low N_2_-fixing capacity, although some effective strains have been identified [49,50]. 

Confirming this information, most of the rhizobial inoculants tested in the recent literature belong to the genus *Rhizobium*, such as *R. etli* [51], *R. leguminosarum* [52], and *R. tropici* [51,53,54,55]. However, inoculation did not always result in a positive response, and in some cases, productivity decreased [54]. For example, Thilakarathna et al. [54] observed a yield variability between −60 and 300% in common beans inoculated with *R. tropici* USDA 9030 and indigenous strains, with an average increase of 140 and 43% in 2015 and 2016 in a humid site (Kaski), and 93 and 8% in 2015 and 2016 in a dry site (Dhading). The evaluation of 39 on-farm trials, inoculation had a success ratio of 2:1 and was especially successful with the introduced USDA 9030 strain. 

Productivity varied between countries, with some producing hundreds of kg [35], while others a few thousand kg per ha [51,53,55,56,57]. For example, Oliveira et al. [51] obtained 1975 kg ha^−1^ with the inoculation of *R. tropici*, which was comparable to the control with 80 kg N ha^−1^. This variation is probably due to local conditions and variations in plant genetic material and the technological levels. For example, in Brazil, experiments were conducted with liming and fertilization and with a high plant density in relatively moderate reliefs, while in Nepal, plants are cultivated in small areas and tutored with a trellis [54].

### 2.3. Mesorhizobium spp.: The Main Microsymbiont for Chickpeas

Turkey and Ethiopia, respectively, are recognized as the primary and secondary centers of chickpea diversity [58]. Turkish landraces spread over the eastern and western Mediterranean and to areas close to Central and Middle Asia, Iran, and Afghanistan. Consequently, they have much higher phenotypic diversity compared to Ethiopian landraces, characterized by a low degree of variability.

Chickpea nodulation is restricted to a few species of microsymbionts, the most common being *Mesorhizobium ciceri* and *Mesorhizobium mediterraneum* [59]. Both macro and micropartners show a certain degree of specificity toward each other, and it is not common for *Mesorhizobium* strains to form cross-nodulation with other legume species. The presence of compatible rhizobial populations is essential for efficient BNF activity, which was not the case at the time of chickpea introduction in western Canada in the early 1990s [60]. The indigenous rhizobial population could not establish symbiosis with the legume species; therefore, nodulation did not occur. In Australia, where all agricultural legumes and their rhizobial associates are exotic, a similar picture was observed when the legume was introduced into the country [61]. These authors showed evidence for horizontal transfer of a mobile gene cluster from the CC1192 inoculant strain of *M. ciceri* to the native Australian rhizobial population. This transfer modified the BNF efficiency of the native population and represented a pivotal contributor to increasing genetic diversity in the resident rhizobial population.

Despite technological improvements such as synthetic fertilizers, pesticides, conventional breeding, and new approaches based on molecular techniques, global chickpea grain productivity is still at the same level as 50 years ago, between 0.5 and 1 tons ha^−1^ [62]. Low efficiency indigenous rhizobial populations, extreme weather conditions, low fertility soils, pathogens, etc., are some factors that severely impact the benefits of BNF to chickpea grain production. Biofertilizers composed of highly efficient nodulating strains are being developed in many parts of the world to maximize atmospheric nitrogen input into farming systems [61]. 

In India, *M. ciceri* is prevalent among chickpea nodule isolates collected at semiarid or subhumid alkaline field sites as determined by a concatenated sequence analysis of six loci [63]. As expected, the isolates did not show cross-nodulation with four other pulses (*P. vulgaris*, *P. sativum*, *L. culinaris,* and *V. mungo*). In the major soil areas of Myanmar, chickpea nodulating rhizobia are most closely related to *Mesorhizobium gobiense*, *Mesorhizobium muleiense*, *Mesorhizobium silamurunense*, *Mesorhizobium tamadayense,* and *Mesorhizobium temperatum* [64]. Around two-thirds of the strains were close to *M. gobiense*, which also represents a common Indian strain CA-181 already mentioned by Singh et al. [63]. In Ethiopia, the secondary center of chickpea diversity, a phylogenetic analysis inferred from the entire genome sequences of 81 rhizobial strains isolated from chickpea nodules harvested from low pH soils showed the presence of eight species of *Mesorhizobium*, as well as a novel species. *Mesorhizobium plurifarium* and *Mesorhizobium loti* represented the two major genomic groups widely spread throughout the sites [65].

Species other than *Mesorhizobium* spp. may also contribute to chickpea nodulation, including *Rhizobium pusense* and *Paraburkholderia kururiensis*, isolated from alluvial soils, and *Rhizobium* sp., *R. tropici*, *Rhizobium multihospitium*, and *Mesorhizobium* sp. from different regions of India [62,66]. 

### 2.4. Bradyrhizobium spp.: The Main Microsymbiont for Cowpeas, Mung Beans, and Pigeon Peas

Cowpea, mung bean and pigeon pea are tropical vegetables considered promiscuous because they are nodulated by different microsymbionts. Strains of the genus *Bradyrhizobium* are the most common, but, depending on the species, the nodules are formed by different species of fast-growing nodulating bacteria.

#### 2.4.1. Cowpeas

Cowpea is considered a promiscuous legume [67]. In this crop, BNF occurs mainly with *Bradyrhizobium* species. However, strains from other genera can be a source of efficient strains for the development of inoculants for this crop [68]. Studies on cowpea inoculation from the last five years are almost restricted to Africa and Brazil. In Brazil, cowpea is an important crop for the Semiarid region and drylands [69]. The Brazilian Ministry of Agriculture, Livestock and Food Supply (MAPA) regulates strains that can be used as commercial inoculants. Concerning cowpea, four strains are recommended: BR 3267 identified as *Bradyrhizobium yuanmingense* [70]; BR 3262 identified as *Bradyrhizobium* *pachyrhizi* [70]; UFLA 03–84 identified as *Bradyrhizobium viridifuturi* [71]; and INPA 3-11B identified as *Bradyrhizobium elkanii* [72]. Inoculation with these strains may significantly increase BNF contributions from 36 to 75 kg N ha^−1^ [69,73,74]. In Venezuela, Artigas Ramirez et al. [75] found that rhizobia nodulating cowpea are mainly fast growing, including the genera *Rhizobium* and *Ensifer,* and the β-rhizobia genera *Burkholderia and Paraburkholderia,* although *Bradyrhizobium* has also been found. In this study, a *Rhizobium* sp. strain showed high symbiotic performance and could be suitable as a novel inoculant for *V. unguiculata* and *V. radiata*. They also found the presence of nitrogen fixation genes from *B. elkanii* in *Paraburkholderia*, suggesting the existence of horizontal gene transfer of symbiotic islands between these species [75]. In a pot experiment, Gyogluu et al. [76] found that *B. elkanii* strains isolated from soybeans in Mozambique were unable to nodulate cowpea, whereas *Bradyrhizobium* sp. strain CB756, a commercial cowpea inoculant strain, was originally isolated from *Macrotyloma africanum* nodules in Zimbabwe [77]. In South Africa, cowpea was nodulated mainly by *Bradyrhizobium* strains, and the legume effectively fixed more than 60% of its total N from the atmosphere in all soil treatments, indicating that *V. unguiculata* may adapt to nutrient-poor ecosystems by establishing a symbiotic interaction with soil native rhizobia [78]. Endophytic, non-rhizobial bacteria such as *Paenibacillus* and *Bacillus* were also isolated. Mbah et al. [79] pointed out the possibly low competitiveness for nodule occupancy by introduced strains, which were highly divergent from native strains obtained from cowpea root or due to their inability to adapt to new environmental conditions in South Africa. Five *Bradyrhizobium* strains isolated in Ethiopia inoculated on five cowpea varieties improved the growth, biomass accumulation, and nodulation performance of the cowpea varieties tested, suggesting that the *Bradyrhizobium* isolates studied can be used to improve cowpea production [80]. Still in Ethiopia, a field experiment showed a significant improvement in seed yield (21%), pod number (16%), and weight of 100 seeds (13%) compared to control in cowpea varieties at three sites and two crop seasons due to inoculation with *Bradyrhizobium* strain CP-24 [81]. 

Quite a few studies focused on studying the natural diversity of cowpea microsymbionts in the last five years [82]. Grönemeyer and Reinhold-Hurek [68] showed a high diversity of *Bradyrhizobium* species in sub-Saharan Africa nodulating cowpea, which appears to be as yet underestimated, and regional strains may be a source to develop adapted inoculants for pulses. Degefu et al. [83], suggest that Ethiopian soils are a hotspot for rhizobial diversity. In Kenya, Nyaga and Njeru [84] demonstrated the existence of effective native rhizobia isolates (*R. tropici*, *Mesorhizobium* sp., and *R. pusense*) in small-holder cowpea farms that improve nodulation, dry shoot weight, and yield. In eastern Kenya, Muindi et al. [85] isolated two native rhizobia (assigned as *Paraburkholderia phenoliruptrix* and *Rhizobium mesosinicum*) with significantly high symbiotic efficiency recorded at 82.5 and 72.8%, respectively, compared to the commercial strain *Bradyrhizobium* sp. USDA 3456 (67.78%). In Senegal, Fatick, Le Quere et al. [86] showed the dominance of *Bradyrhizobium vignae* as a primary symbiont of cowpea. 

#### 2.4.2. Mung Beans

*Bradyrhizobium* is the predominant mung bean symbiont in Brazilian agricultural sites, and among the species, *B. yuanmingense* promotes the greatest increase in shoot biomass [87]. *B. yuanmingense* is also one of the species inhabiting mung bean nodules in Pakistan [88]. This genus has been demonstrated as promoting significant increases in nodulation and root and shoot biomass, and strains of the *Bradyrhizobium japonicum* superclade were shown to be better growth promoters than strains of the *B. elkanii* superclade [89]. *Bradyrhizobium* has also been found nodulating mung beans in Ethiopia, which can be considered as a hotspot for rhizobia diversity [83]. In this country, *Bradyrhizobium* inoculation associated with P fertilization increased BNF and N yields in a low-input area, and it may be an alternative to increase grain yield in small-holder farmers. However, this genus is not dominant in all cases. For example, a co-dominance between *Bradyrhizobium* and *Ensifer* was observed in Pakistan, while in the rhizosphere they represent only a small fraction associated with the phyla Proteobacteria [90]. 

In Brazilian tropical soils, mung bean nodulation with native rhizobia is lower in the sampled areas of the Cerrado, the Brazilian savannah, compared to the Atlantic Forest [87]. On the other hand, in Australia, the *Bradyrhizobium* strain CB1015 from India is recommended for inoculation of mung beans [91], but it is rarely detected in agricultural areas, including inoculated sites [92]. The authors suggest that indigenous strains may be more competitive than the inoculant strain in some environments. In fact, they found that some wild bradyrhizobia capable of forming nodules in mung beans may be as effective as commercial inoculum. 

Cross-nodulation with different *Bradyrhizobium* strains has also been observed. Three out of four *Bradyrhizobium* strains used in Brazil as a commercial inoculant for cowpea, *B. viridifuturi*, *B. yuanmingense*, and *B. elkanii,* successfully nodulate mung bean, but not *B. pachyrhizi* [93]. These results indicate the existence of some level of incompatibility between strains of the *Bradyrhizobium* genus in relation to the colonization of mung bean nodule colonization. A type III secretion system (T3SS) is responsible for the interaction between mung bean and *Bradyrhizobium* strains through T3SS mutations that determine cultivar specificities and nodulation properties. The USDA 61 strain of *B. elkanii* is not compatible with mung bean and soybean due to the presence of a functional T3SS. As a result, it does not nodulate with either plant [94]. In addition to T3SS, an *innB* gene may also restrict nodulation and control symbiosis with *Vigna* species. In contrast, Piromyou et al. [95] evaluating four *Bradyrhizobium* strains that share a common origin based on similarities in T3SS, found that the bradyrhizobial strain SUTN9-2 showed the best interaction with mung bean, due to its specific T3SS.

Furthermore, several other genera can be isolated from mung bean nodules, such as *Bradyrhizobium*, *Rhizobium*, *Mesorhizobium*, *Ensifer*, *Leifsonia*, *Bacillus*, *Agrobacterium*, *Mycolicibacterium*, and *Kaistia* [87]. *Leifsonia*, *Bacillus*, *Agrobacterium*, *Mycolicibacterium*, and *Kaistia* are non-rhizobial bacteria and can be considered as nodule endophytes. Hakim et al. [88] found, in addition to *Bradyrhizobium* strains, the fast-grower species *Ensifer aridi* and *Ensifer meliloti*, as well as *R. pusense*, all capable of establishing effective symbiosis with mung bean [88].

Inoculation of mung beans with rhizobial strains that have PGPR traits may result in greater symbiotic efficiency. IAA production and P solubilization ability are present in three rhizobial isolates from mung bean nodules [96]. The isolate assigned to *B. elkanii* showed the best PGPR characteristics and, as a result, had the highest nodulation and shoot biomass under axenic conditions. A strain of *R. pusense*, an endophytic bacteria isolated from mung bean roots, has several PGPR traits: siderophore, IAA, and ammonia production; and ACC deaminase and P solubilization activities [97]. Additionally, proteins and transporters related to stress tolerance are also present. In a pot experiment, this strain promoted a substantial increase in nodulation and shoot biomass. In another study, a rhizobial strain that is capable of producing bacteriocin was co-inoculated with the recommended rhizobial strain and resulted in significant increases in nodulation, root and shoot biomasses, chlorophyll and leghemoglobin content, and grain yield, among other factors, compared to inoculation with single-rhizobial inoculation [98]. There is an increase in nodular occupation by the bacteriocin-producing strain, which may be a strategy for the development of promising biofertilizers.

#### 2.4.3. Pigeon Peas

Nodulation in pigeon pea roots is often poor, and understanding of symbiotic efficiency is still limited. It is crucial to study the pigeon pea microsymbiont to maximize the contribution of BNF to increase plant growth and grain productivity. The main factor influencing the native population capable of forming root nodules with pigeon peas, according to Chalasani et al. [99], is the crop area, followed by the plant developmental stage and soil type. In their study, plant genotypes play a small role, while for Bopape et al. [100], data from the analysis of forty soil samples showed a differential N fixation ability among them, which is mainly dependent on the host plant genotype.

Pigeon peas are nodulated by a wide range of rhizobia comprising *Bradyrhizobium* and fast-growing rhizobia. It is highly tolerant to drought, although the yields tend to reduce under these conditions. BNF through inoculation with elite strains characterized by effective symbiotic traits is a strategy to improve crop yield. Nineteen strains isolated from different soil types in India and selected from nodulation and productivity data were submitted to complete genome sequencing [101]. Jorrin et al. [101] found that these strains belong to *Bradyrhizobium* and *Ensifer*, and *B. yuanmingense* was the most common species. In terms of productivity, strains of *B. yuanmingense* and one strain of *Ensifer alkalisoli* were the best available resources for inoculant development. However, nodules from pigeon pea, chickpea, and sweet pea (*Lathyrus sativus* L.) growing in Bangladesh were mostly colonized by a *Rhizobium* sp., which promoted increased nodulation and shoot dry weight in the three species, also being a potential inoculant for pigeon peas.

Two classical strains used for soybean inoculation were tested in pigeon pea: a fast-growing strain, *Ensifer fredii* (USDA 191), and a slow-growing strain, *Bradyrhizobium diazoefficiens* (USDA110), in addition to the respective T3SS mutants [102]. All inoculants produced Fix+ nodules, except USDA 110, which did not have rhizobia or leghemoglobin in its interior. Furthermore, nodulation assays with the USDA 110 strain T3SS mutant showed high competitiveness, together with the ability to fix atmospheric nitrogen. Despite promising results, more studies are needed.

## 3. PGPR Benefits: Nutrition Enhancement and Tolerance to Abiotic Stresses

Research on PGPR is abundant, which reflects the interest and the importance that microorganisms have for plant development. These microorganisms establish themselves in the rhizosphere, root, and shoot where they act through multiple mechanisms. Among them are: (1) the production of plant growth regulators; (2) increased root absorption (formation of adventitious roots and absorbent hairs, membrane transporters, stimulus to the proton pump); (3) availability of nutrients in the soil solution (solubilization of potassium and phosphate rocks, production of organic acids, production of siderophores, exopolysaccharides, and hydrolytic enzymes—proteases, pectinases, lipases, and chitinases and phytases); (4) mitigation of the effects of abiotic stresses (production of ACC deaminase and exopolysaccharides, and stimulus to the accumulation of antioxidant enzymes—catalase, peroxidase, polyphenol oxidase, superoxide dismutase and ascorbate peroxidase, and of non-enzymatic antioxidant metabolites—glutathione and ascorbic acid). Growth promotion also occurs indirectly through the relationships of hyperparasitism, antagonism, competition, and the induction of resistance in plants to phytopathogens. Research on PGPR is abundant, which reflects the interest and the importance that microorganisms have for plant development [103,104,105,106,107,108,109,110].

In this section, assays with approximately 160 microorganisms focused on pulse growth promotion were described, totaling 81 taxa. Most of these studies consist of selecting microorganisms capable of producing specific applications in plants by improving various nutritional aspects and increasing tolerance to climatic stresses. The results point to an increase in chlorophyll levels, nutrients such as N, P, K, Ca, Mg, K, S, proteins, and sugars in plant tissues; leaf area; shoot and root biomass; grain yield, grain weight, number of pods per plant, and harvest index; and reduction in plant disease incidence. The translation of these results into technologies applied to agricultural production systems aims to increase productivity and replace or reduce the use of soluble chemical fertilizers. In addition, a greater number of studies related to the culture of common beans, chickpeas, peas, broad beans, cowpea, and mung bean were found.

Table 2 shows the seven most common taxa used in studies as PGPR. *Bacillus* sp. and *Pseudomonas* sp., including *B. subtilis* and *P. fluorescens,* have been tested in the eight leguminous pulses. A broader look at the interaction of microorganisms with pulses suggests, on the one hand, potential promising groups for the development of biotechnological applications and, on the other, little-studied groups that perhaps deserve greater attention.

### 3.1. Temperate Pulses

#### 3.1.1. Chickpeas

Co-inoculation of *Mesorhizobium* with PGPR has been considered as a promising strategy for chickpea inoculation. A consortium of a *Mesorhizobium* strain used in India with *Pseudomonas fluorescens* and *Pseudomonas argentinensis* isolated from the rhizosphere and roots of wild chickpeas improved symbiotic traits, soil quality, and grain yield compared to the single *Mesorhizobium* inoculant [128]. The authors consider the consortium to be a potential biofertilizer for promoting sustainable agriculture. Co-inoculation of actinobacterial endophytes isolated from chickpea roots with *M. ciceri* increased total plant N and approximately 30% of shoot biomass [155,156]. Exudates from chickpea roots colonized by actinobacteria stimulate nodulation-related biological processes and may help improve chickpea production under field conditions.

Phosphate (P) and zinc (Zn) solubilizers are important PGPR traits essential to increase BNF, plant growth, and yield. A P-solubilizing microbial consortium that included strains of *Proteus mirabilis*, *Pseudomonas* sp., *Pseudomonas aeruginosa*, *Chryseobacterium* sp., and *Klebsiella pneumoniae* was isolated from a lab-scale bioreactor [113]. The consortium significantly improved growth and reduces fertilizers by 50–100% in both chickpea and mung bean. Two P- and Zn-solubilizing bacterial strains of the *Bacillus* and *Pseudomonas* genera isolated from the rhizospheric soil of a chickpea production area promoted an increase of approximately 17% in grain yield [111]. In areas with a low rhizobium population, the addition of *Mesorhizobium* to the P- and Zn-solubilizing strains was necessary. A Zn-solubilizing strain of *Pseudomonas protegens*, which also possess P solubilization and ACC deaminase activities, promoted an approximately 40% increase in shoot length compared to control plants [157].

Arbuscular mycorrhizal fungi (AMF) were also successfully used as a biofertilizer for chickpea. Under field conditions, multiple AMF isolates applied by seed coating stimulate mycorrhizal root colonization and increase grain yield by 140% compared to a single AMF isolate [144]. Comparing AMF symbiosis with non-AM fungal endophytes, Bazghaleh et al. [158] showed that the former increased chickpea biomass, while the latter had a neutral effect. The effect is cultivar-dependent, and co-inoculation of AMF and non-AM fungal endophytes may show different responses.

Chickpea in arid and semi-arid environments is frequently affected by harsh environmental stresses such as heat, drought, and salinity, which limit its growth and productivity, and are aggravated by climate change. Enriching microbial colonization can help alleviate stressful conditions. Small noncoding regulatory RNA molecules, the microRNAs, are modulated by bacteria during various environmental stresses. *Pseudomonas putida* promotes a variable expression pattern of individual microRNAs and their target genes in a tolerant chickpea genotype exposed to drought and salt stress [159]. The authors concluded that specific miRNA-mediated perception and response mechanisms operate under these stresses. ACC deaminase activity may improve tolerance in salinity and drought situations. *Azotobacter chroococcum*, *B. subtilis*, *P. aeruginosa*, and *Bacillus pumilus*, all of them with a high level of ACC-deaminase activity, endured different pH, temperature, and NaCl concentrations [137]. The consortium with the four strains exerted a positive impact on the growth of chickpea plants under normal conditions compared to uninoculated plants.

Regarding salinity stress, chickpea seed inoculation with rhizobium improves mineral uptake, reduces electrolyte leakage, which directly influences photosynthesis, and increases yield attributes in salt-treated plants [160]. A consortium composed of *Bacillus safensis*, *Pseudomonas stutzeri*, and *Staphylococcus xylosus* increases the total fresh weight of chickpea under salinity stress by 54% in a field experiment, compared to the control under normal conditions [161].

Chickpea co-inoculation of *M. ciceri* and *P. fluorescens* significantly improved the fresh and dry weight of leaves, roots, and shoots under drought stress conditions, although it did not alter the nodulation parameters [129]. Laranjeira et al. [112] inoculated chickpea seeds with bacteria (*Mesorhizobium* sp., *Burkholderia* sp., and *Pseudomonas* sp.) and AMF (*Rhizophagus irregularis*, *Funneliformis geosporum*, and *Claroideoglomus claroideum*). Under field conditions, irrigated only during reproductive stages, they observed the highest cumulative grain yield (18,157 kg ha^−1^), resulting in an increase of 16% and 237% over fully irrigated inoculated plants and non-inoculated plants under rainfed conditions, respectively.

#### 3.1.2. Peas

Pea is one of the first domesticated crops, and it contributes to sustainable agriculture by playing important agronomic, economic, and environmental roles [27]. It is an alternative cover crop in semi-arid regions, capable of generating an economic return due to grain production [27]. The co-inoculation between PGPR and the Rlv strain has shown some promising results in pea. Mamontova et al. [145] observed that the co-inoculation of pea line K-8274 with Rlv and AMF strains resulted in a significant and stable increase in shoot and seed biomass compared to line K-3358. K-8274 showed a high efficiency of interaction with soil microorganisms (EIBSM) in comparison to a low-EIBSM line K-3358. Although, the molecular mechanisms behind this effect are not fully understood, the responsive line, K-8274, exhibited prolongation of seed maturation by up-regulation of proteins associated to cellular respiration and protein biosynthesis, and down-regulation of proteins during late-embryogenesis, while the low-EIBSM line K-3358 displayed lower levels of the proteins related to cell metabolism. The authors suggested that EIBSM trait should be considered in pulse breeding programs. In a soil-filled pot experiment, co-inoculation of pea seeds with Rlv and AMF strains followed three inoculation schedules and resulted in an increase of approximately 30% in seed yield compared to the control inoculated with the Rlv strain only [147]. In addition, co-inoculation increased ascorbic acid, protein, and carbohydrate contents in the seeds, while no significant differences occurred between pre-sowing or post-emergence applications. 

To evaluate the response of biofertilizers in an agroforestry system, Shukla et al. [152] co-inoculated several legumes in the spring and winter with specific rhizobial strains for each pulse, a PSB strain, and two AMF species, *Acaulospora scrobiculata* and *R. irregularis*, common to all crops. Grain yields, rhizobial nodulation, and AMF colonization were lower in the shade compared to plants growing in full sunlight. Consortium inoculations were effective in peas, chickpeas, lentils, and mung beans, among other crops, under both light conditions, suggesting that their use in agroforestry systems may at least partially overcome the adverse effect of shading typical of this type of system [152].

In a three-year field experiment, two consortia formed by a Rlv strain and either a (1) P-mobilizing strain, *Lelliottia nimipressuralis*, and *Paenibacillus polymyxa* carrying phytopathogen antagonistic properties, or (2) a phototrophic N_2_-fixing strain of *Nostoc linckia* significantly increased pea seed yield [162]. The three years average results compared to the mineral fertilizer treatment were heavily influenced by the climatic conditions. The yields range from around 2.1 to 3.5 tons ha^−1^, the former observed under low soil, low air humidity, and low rainfall, but the positive results derived from the inoculation of the consortia were independent of the climate conditions.

PGPR strains may also be used as a biofertilizer for pea cultivation. *Pseudomonas*, *Bacillus*, and *Sanguibacter inulinus* isolated from the rhizospheres of rapeseed, winter pea, and faba bean showed a high proportion of P solubilization, but the abilities of PSB strains isolated from rapeseed were significantly higher than those isolated from winter pea and faba bean [114]. The authors emphasized the influence of the plant on its microbiome to fit P demand. 

Pea plants are highly sensitive to salinity stress, which can be minimized by inoculation with different microorganisms. *Acinetobacter bereziniae*, *Enterobacter ludwigii*, and *Alcaligenes faecalis* strains possess various PGPR traits (IAA, siderophore, and exopolysaccharide production, P, Zn, and K solubilization, and ACC deaminase activity); they are salt-tolerant and can mitigate salinity stress [163]. The three of them enhanced the growth parameters of pea seedlings by improving antioxidant enzyme activity associated with salt stress alleviation, but *A. faecalis* showed the best performance. Furthermore, in field trials, the authors observed that inoculation increased pea growth and grain yield in the presence of NaCl stress (100 mM). The *Bacillus marisflavi* and *Bacillus cereus* strains selected for their potent ACC-deaminase activity were able to mitigate symptoms and increase plant biomass and levels of antioxidant enzymes, among other parameters, when inoculated in pea plants under NaCl stress [124]. Other *Bacillus* strains, *B. subtilis*, *B. safensis*, and *B. cereus* selected for their PGPR traits (IAA, P solubilization, siderophore, and ammonia production) were further evaluated on pea seedlings under 1% NaCl stress [138]. In addition to improving plant growth parameters, inoculation also increases antioxidant enzyme activities, preventing oxidative damage caused by reactive oxygen species (ROS).

Inoculation with a strain of halotolerant *Kocuria rhizophila*, a Gram-positive bacterium that belongs to the order *Actinomycetales*, also improved growth and oxidative enzyme activities in pea plants subjected to saline stress. The authors point out that the response was defined by a fine interaction between *K. rhizophila* and pea genotypes modulated through the antioxidant system [164].

A consortium inoculation with strains from different families of AMF, *Rhizophagus fasciculatus*, and *Gigaspora* sp. benefits pea production under salinity stress by reducing adverse effects through the improvement of the antioxidant system [146]. The AMF consortium exhibits a better response compared to a single AMF inoculation. The authors considered that incompatibility between symbionts might explain the difference between AMF treatments [165].

In addition to salinity stress, bacteria containing ACC-deaminase may also mitigate drought stress in peas. Inoculation with Rlv, which generally has ACC deaminase activity, may enhance shoot biomass, nodulation, and BNF activity of pea plants subjected to water-deficit stress [166]. A consortium composed of three strains of rhizobacteria producing ACC-deaminase, *Ochrobactrum pseudogrignonense*, *Pseudomonas*, and *B. subtilis* significantly increases the percentage of seed germination, the lengths of the roots and shoots, and the dry weight of inoculated plants exposed to drought stress [115].

Most of the authors suggested that these strains may be used to mitigate abiotic stress, contributing to the maintenance of plant health and to sustainable crop development.

#### 3.1.3. Lentils

Field experiments under rainfed and irrigated conditions were carried out in Iran with lentils inoculated with *Azotobacter*, a soil N_2_-fixing bacteria, and *Glomus intraradices*, an AMF species [167]. Respectively, yield and seed protein were 6.5 and 20.5% higher under rainfed conditions and 21.9 and 28.2% under irrigated conditions compared to the control. The highest seed yields, 649.03 kg ha^−1^, and seed protein, 159.77 kg ha^−1^, were produced under supplemental irrigation and inoculation with AMF and *Azotobacter*. 

Lentil biofertilization with *R. leguminosarum* and/or *P. fluorescens* was evaluated at different levels of P [168]. The application of 40 kg P_2_O_5_ resulted in maximum uptake of N, P, and K. Using similar treatments, Singh et al. [169] observed that lentil co-inoculation with *Rhizobium* and *P. fluorescens* promoted significantly higher grain yield after receiving 20 kg P_2_O_5_ ha^−1^ than after receiving 40 kg P_2_O_5_ ha^−1^ without biofertilizer. 

The agroforestry system is an alternative for managing multiple crops, with natural competition for plants for nutrients, water, and light, as mentioned by Shukla et al. [152] in the previous section. 

Sixty-three rhizobacteria isolated from lentils were obtained from nine soils in the Mediterranean area [116]. Ten selected strains were identified as *Pseudomonas* spp. One of them, close to *Pseudomonas umsongensis*, increased early nodulation by 85% when co-inoculated with rhizobium compared to single-rhizobial inoculation. This strain showed higher ACC-deaminase activity and IAA production. No statistical differences were found (*p* < 0.05)].

The use of an N_2_-fixing *Rhizobium* with P-mobilizing microorganisms (*Priestia megaterium* and *P. polymyxa*) increased lentil yield [170]. An additional increase of approximately 8% in grain yield was obtained when a seaweed extract biostimulant was also applied. 

Besides *Azotobacter*, *Pseudomonas*, *Paenibacillus*, *Enterobacter*, and *Bacillus* are commonly used as plant growth-promoting agents and can be of potential interest for agricultural applications under climate stress conditions. Their combination with rhizobia has shown synergistic growth and nodulation outcomes. An irrigation assay was conducted at 30 and 50% levels to assess the maximum water-retaining soil capacity. Lentil co-inoculation with *Rhizobium* and PGPR under sufficient watering conditions (50%), showed a significant increase in shoot dry weight for several strain combinations: *R. laguerreae* and *Enterobacter aerogenes*, *R. laguerreae* and *Bacillus* sp., *R. laguerreae* and *Bacillus* sp. as well as the single inoculation of *E. aerogenes*, compared to the nitrogen fertilizer control [125]. Shoot dry weight decreased under water stress (30%) compared to normal watering treatment. Generally, all consortium combinations increased shoot biomass compared to single inoculations and the fertilized control, except the consortium composed of *R. laguerreae* and *Bacillus*.

Growth and yield can be significantly improved by co-application of ACC-deaminase-producing rhizobacteria (*Bacillus amyloliquefaciens* and *Lysinibacillus fusiform*) and caffeic acid under drought stress [171]. However, the effect of *B. amyloliquefaciens* was more prominent than *L. fusiform* for most of the growth attributes of lentils when caffeic acid was added under drought stress. Caffeic acid is converted to ferulic acid under drought stress through the O-methyltransferase enzyme. Both ferulic acid and caffeic acid are accumulated in the leaves during drought stress conditions. These antioxidants attach to the leaf cell wall and protect the photosynthetic apparatus from high-energy radiations by absorbing them into mesophyll cells [172]. Caffeic acid and its derivatives are important in enhancing nitrogen fixation, and carbohydrates and protein content in nodules [173].

Cytokinins are another important group of growth regulators responsible for cell division, nutrient allocation, and photosynthetic performance. A *Methylobacterium oryza* cepa was selected based on its high cytokinin production and its tolerance to water stress tested by in vitro exposure of lentils to PEG6000 [174]. The presence of *Methylobacterium* significantly improved the performance of lentils exposed to drought by: a. stimulating the initial growth of shoots and roots; b. increasing photosynthetic rates for well-irrigated and water-stressed conditions; c. improving the harvest index by seven times for well-watered lentils and four times for drought-stressed plants. 

#### 3.1.4. Broad Beans

In a field experiment for two consecutive years, broad beans co-inoculated with rhizobia and *P. fluorescens* at 10^9^ CFU ml^−1^ extended the period to reach the maximum leaf area and increased grain yield and dry shoot biomass [130]. The environmental conditions influenced the results. Co-inoculation gave the best results in the first year, while in the second year, when unfavorable conditions were found, a single inoculation with *Pseudomonas* was the best treatment. Rhizobial inoculation can promote N exudation to the rhizosphere, promoting an increase in PGPR populations, which also contributes to stimulate nodulation and root growth [36].

In a greenhouse, broad bean was inoculated with an *A. chroococcum* isolate capable of producing several hydrolytic enzymes. Inoculation showed higher and lower values for shoot and root biomasses, compared to the uninoculated control and NPK fertilization treatment, respectively [142]. *A. chroococcum* inoculation may be an alternative capable of replacing, at least partially, mineral fertilization by increasing the efficiency of the fertilizer and reducing costs.

Co-inoculation of broad beans with *Rhizobium* sp. isolated from nodules of faba beans and *Rahnella aquatilis* or *Pseudomonas brassicacearum*, both isolated from the rhizosphere of broad beans, significantly increased shoot and pod biomass and P content, in addition to increasing root phosphatase-phytase activities [175]. In a field experiment in Morocco, PGPR strains isolated from the broad bean rhizosphere (*R. aquatilis* and *Acinetobacter pittii*) and an *E. meliloti* strain were inoculated as a single strain or in a mixture on broad bean and *Triticum durum* (wheat) plants [176]. Shoot and root dry weight more than doubled compared to the uninoculated control whether single-rhizobial strain or its mixture with PGPR were used. The results suggest that the growth stimulus was mainly due to BNF. The authors concluded that the best responses to inoculation, associated with increased growth and nutrient absorption, occurred with the mixture of PGPR and when *V. faba* was intercropped with *T. durum*. The introduced PGPR strains can probably establish and colonize the rhizosphere of both species, promoting growth in single cultivation and with a synergistic effect in intercropping.

The inoculation of broad bean with AMF reveals promising results that have been motivating the execution of several studies. Rakiami et al. [177] inoculated broad beans under field conditions cultivated in a calcareous soil with pH 7 in Morocco with a tripartite biofertilizer composed of two PGPR strains *Acinetobacter* sp. and *R. aquatilis*, two strains of *E. meliloti,* and the AMF species, *Glomus* sp., *Sclerocystis* sp., and *Acaulospora* sp. Increases in shoot and root biomasses, pod weight and number, and shoot content of N, P, Ca, K, Na, sugars, and proteins were shown. *Rhizobium* inoculation was sufficient to improve the N content, while mycorrhizal fungi expanded the frequency of root mycorrhization (>90%). However, broad bean inoculation with rhizobia, *R. laguerreae*, and AMF (*R. irregularis*, *F. geosporum*, and *C. claroideum*) does not show a uniform response in terms of increases in nodulation and BNF levels [153]. The number of nodules decreased and single-rhizobial inoculation stimulated the mycorrhizal colonization rate. Sanchez-Navarro et al. [154] cultivated broad beans in a semi-arid area in Spain with a complex mixture of microorganisms, made up of *R. leguminosarum*, *Burkholderia cenocepacia*, *Burkholderia vietnamiensis*, and the AMF species *R. irregularis*, *Claroideoglomus etunicatum*, *C. claroideum*, and *Funneliformis mosseae*. The authors found that single inoculation with *R. leguminosarum* led to an increase in seed N and protein content, although it did not increase BNF. AMF inoculation increased N levels in seeds, shoots, and roots, probably due to efficiency of nutrient use.

In addition to the availability of P in the soil, micronutrients can improve the effect of broad bean inoculation with AMF. El-Mansy et al. [148] reported the beneficial effect of Fe foliar fertilization (400–500 ppm) in a two-year field experiment in arid regions of Egypt, where reduced Fe availability is common due to alkaline pH. The addition of Fe addition increased AMF colonization, the native and inoculated number of AMF spores, and the number of active nodule number. The synergistic effect between AMF inoculation and Fe addition produced a 100% increase in crop yield. In contrast, the combined effect of the application of Zn through soil (5 mg kg^−1^ soil) and AMF inoculation in broad beans cultivated in sterilized and non-sterilized soil in a greenhouse showed that Zn fertilization did not influence mycorrhizal colonization, while increases in both dry shoot biomass and nutrient concentrations in tissues of inoculated plants were observed, regardless of mineral fertilization [149].

*Ensifer saheli*, an endophytic isolate from the nodules of a tree legume from the Aswan Desert in Egypt, was able to reduce the light irradiance stress in broad bean seedlings in a greenhouse experiment. The strain, in addition to fixing N, produces hydrolytic enzymes and IAA, promoted greater efficiency of water use, reduced stomatal conductance, and increased chlorophyll and protein content, length, and root and shoot biomasses [178].

Under drought stress, the co-inoculation of Rlv and *P. putida* shows the best results compared to isolated strains, such as increased root and shoot parameters, seed production, and water use efficiency [179]. The authors suggested that the ACC deaminase enzyme produced by *Pseudomonas* reduced the ACC, precursor of ethylene in plants, and stimulated greater absorption of nutrients and induced the formation of absorptive roots.

A single inoculation or a co-inoculation with halotolerant *B. subtilis* and *Bacillus thurigiensis*, both isolated from halophytic plants in Egypt, mitigated the effect of salt stress by promoting the growth of shoots and roots, and increasing the absorption of minerals such as N, P, K, Ca, Mg, and K [139]. Broad bean inoculation with *R. leguminosarum* combined with nonnodulating exopolysaccharide-producing strains such as *Paenibacillus mucilaginosus* and *E. meliloti* improved the plant’s tolerance to salt stress, suggesting a direct or indirect effect on plant response. Indirectly, growth promotion was related to the modulation of soil microbiota adhered to the roots.

Three strains of *Pseudomonas* isolated from saline soils and vineyards in Algeria were selected for their ability to grow in culture medium with sodium chloride (7%), produce indole acetic acid, and siderophore pyoverdine [180]. Broad bean inoculation significantly increased fresh biomass production in the presence or absence of salt stress in a greenhouse experiment. 

### 3.2. Tropical Pulses

#### 3.2.1. Common Beans

The recent literature cites the co-inoculation of common beans with rhizobia and several other bacteria, such as *Azospirillum*, *Bacillus*, *Paenibacillus*, *Pseudomonas*, *Cyanobacteria*, and *Burkholderia*, among others [133,181,182,183,184]. A classic example is the co-inoculation with *Rhizobium* and *Azospirillum*, which has been reported to increase yields with return rates between 90% and 114% in commercial farming [181]. De Carvalho et al. [185] found that co-inoculation of *R. tropici* with *Azospirillum baldaniorum* or *B. elkanii* 29w contributed to early nodulation and biomass accumulation in common beans. Additionally, in a field experiment, Leite et al. [55] observed that the co-inoculation of *R. tropici* with *B. diazoefficiens* CPAC 7 contributed to an increase of approximately 20% in yield compared to the single inoculation of *R. tropici*, with a yield greater than 3000 kg ha^−1^. Finally, Pastor-Bueis et al. [143] evaluated the efficacy of the inoculant based on the elite strain *R. leguminosarum* bv. *phaseoli* LCS0306 (R), looking for the optimal combination with *P. brassicacearum* subsp. *neoaurantiaca* (P) and *A. chroococcum*. The consortium between *Rhizobium* and *Pseudomonas* increased the contribution of N_2_ fixation by 51.7 kg ha^−1^ (87 %) and the yield by 1337 kg ha^−1^ (59%) compared to the control without inoculation and without fertilization.

The success of co-inoculation may be due to a complex combination of several factors, such as the ability of the non-rhizobial strain to colonize the interior of the nodule, located in a separate niche, which is the case of *Pseudomonas*, thus enabling action as a PGPR without competition with its rhizobial partner. Several bacterial genera such as *Pantoea*, *Klebsiella*, *Rhizobium*, *Enterobacter*, and *Bacillus* have been reported as nodule endophytes, either by culture-dependent and independent methods [43,126]. Additionally, other diazotrophic bacteria, such as *Azospirillum*, establish less direct relationships with the host plant but are also capable of supplying, at least partially, the plant’s N demands [186,187]. In the case of the co-inoculation reported by Leite et al. [55], the *Bradyrhizobium* strains induce ineffective nodulation when singly inoculated. However, a synergistic effect is obtained when they are co-inoculated with *R. tropici*. The mechanisms behind this interaction remain to be unveiled.

Regarding P absorption, the association between mycorrhizal fungi and the common bean represents a viable alternative. BNF is an energy-demanding process, and adequate provision of P to legumes contributes to improving the efficiency of the symbiosis. AMF are the key in this regard, since they help plants absorb nutrients, especially P, in addition to contributing to water and improving resistance to environmental stress [188]. In common beans, Razakatiana et al. [189] evaluated the effects of inoculation of AMF isolates identified as *Acaulospora* sp. and *Glomus* sp. together with a group of ten *Rhizobium* strains, reporting a synergistic effect of double inoculation on plant P content, nodulation, mycorrhization rate, and acid phosphatase activity. The plant’s P content, which was 0.85 g kg^−1^ without inoculation, increased to 1.57 g kg^−1^ with double inoculation, corresponding to an amount three times higher of P accumulated in the plant biomass. Likewise, the mycorrhization rate also increased, 30–63% for inoculation with mycorrhiza only, and 80–95% for inoculation with mycorrhiza and rhizobia; that is, an increase of 28–216%.

Microbial inoculants can also help mitigate the effects of biotic and abiotic stresses, such as salinity and temperature [190]. This is relevant since the common bean is sensitive to high and low temperatures as well as to thermal and saline stress. *Bacillus* spp. were demonstrated to enhance several parameters under osmotic and thermal stresses either alone or when co-inoculated with other microbes such as *Pseudomonas* and AMF. Inoculation with this genus improved seed germination and plant growth and reduced oxidative osmotic stress, and the effects were strain-dependent [117]. Benefits were also observed when *B. subtilis* was combined with *P. fluorescens* and silicon, contributing to bean growth, yield, and biochemical parameters, such as catalase and superoxide dismutase activities [132]. In another experiment, the co-inoculation of *B. amyloliquefaciens* and AMF benefited the photosynthetic and transpiration rates, stomatal conductance, and yield of common beans under water stress. Finally, de Lima et al. [140] observed that non-inoculated plants showed lower growth when exposed to high temperature (35 °C against 25 °C in the control) compared to plants inoculated with *B. subtilis*, which increased shoot biomass (40%), shoot:root ratio (30%), and number of leaves (25%) in plants subjected to thermal stress during the reproductive phase. Similarly, bacteria can also help plants cope with the stress caused by low temperature. Psychrotolerant bacteria, among them four species of *Pseudomonas* and one *Brevibacterium frigoritolerans*, decreased freezing injury, ice nucleating activity, lipid peroxidation, and stimulated antioxidant enzyme activity, contributing to cold tolerance of inoculated common beans [191].

According to Talaat et al. [192] the application of effective microorganisms (EM) showed protective roles against stress and a mechanism of protection of the photosynthetic apparatus of bean plants was found. They helped maintain photosynthetic pigments, improved PS I and PS II activities, improved gas exchange parameters, regulated chlorophyll fluorescence kinetics, and induced Rubisco activities.

#### 3.2.2. Cowpeas

PGPR may help legume–rhizobia symbiosis and improve cowpea productivity. Jayakumar et al. [120] inoculated *Pseudomonas* spp. in cowpea seeds and saw a significant increase in growth parameters such as shoot length, root length, and root numbers in inoculated plants compared to the control. Valdez-Nunez et al. [193] isolated bacterial strains from healthy cowpea root nodules capable of solubilizing tricalcium phosphate, producing siderophores, and with antagonistic activity against *Fusarium oxysporum*. Regarding this, the co-inoculation of efficient *Bradyrhizobium* strains [194], with *Azospirillum brasilense* [194,195,196,197], AMF [78,150], *Trichoderma* [198,199] and P-solubilizing fungi [200] may help cowpea plants absorb more nutrients from soil and increase grain yield. 

Through culture-independent molecular methods, Le Quere et al. [86] accessed the diversity of bradyrhizobial populations in association with cowpea nodules in Senegal and found that *Bradyrhizobium vignae* was the dominant symbiont. Puozaa et al. [201] detected the presence of highly diverse bradyrhizobia (i.e., *B. vignae, B. elkanii*, *B. iriomotense, B. pachyrhizi*, *and B. yuanmingense*) and also unidentified bradyrhizobia in acid soils from Ghana and South Africa. Mukhtar et al. [118] evaluated the diversity of DNA isolated from cowpea nodules in Pakistan and the dominant bacterial genus in the nodule microbiomes was the α-proteobacterial genus *Bradyrhizobium*, but other proteobacterial genera, some distinct from rhizobia such as *Acidiphilium* and *Pseudomonas*, were also detected. Bacterial isolates from cowpea nodules from the same soils were identified and often shown to be PGPR, namely *Mesorhizobium, Ensifer, Bradyrhizobium, Paenibacillus, Bacillus, Pseudomonas*, and the actinobacteria *Frankia* sp., *Streptomyces galilaeus*, and *Streptomyces griseoaurantiacus*. Some isolates also showed plant growth-promoting traits such as nitrogen fixation, P solubilization, and siderophore and HCN production. Antifungal activity and extracellular enzymes such as cellulase, lipase, chitinase, amylase, and protease were also detected. Stress-tolerant bacteria may also improve cowpea growth under salinity stress conditions [119]. Abiala and Sahoo [202] found that *Bacillus filamentosus* (C8) and *Bacillus aryabhattai* (C29) were able to protect cowpea under NaCl-induced salinity stress, probably due to stabilized membrane and enhanced proline content.

#### 3.2.3. Mung Beans

Yousefi et al. [203] evaluated leaf area index and radiation use efficiency in mung beans in six fertilization treatments: (1) free-living N_2_-fixing bacteria, (2) P-solubilizing bacteria, (3) K-solubilizing bacteria, (4) a mixture of N_2_-fixing, P-solubilizing and K-solubilizing bacteria, (5) nitrogen fertilizer, and (6) control (without biological or chemical fertilizers). Tripartite inoculation was the best treatment considering the parameters evaluated. 

*Pseudomonas*, *Bacillus*, and *Acinetobacter* isolated from mung bean rhizosphere produced under in vitro conditions IAA (from 45.66 to 111.94 µg mL^−1^) and siderophore, in addition to possessing P solubilizing (from 952.91 to 1341.24 µg mL^−1^) and catalase activities [122].

Bilal et al. [121] investigated the effect of *Pseudomonas* spp., a PSB, as inoculant for mung bean varieties, and reveal a significant improvement in grain yield of 882.23 kg ha^−1^, similar to 70 kg ha^−1^ of P fertilization, greater than the control. Other parameters such as root and shoot dry weight, root and shoot length, weight of 1000 grains, and harvest index also improve with *Pseudomonas* inoculation.

Mung bean inoculation with strains of *P. aeruginosa* or *B. subtilis*, both isolated from the rhizospheric soil of healthy mung bean plants, promoted an increase in shoot and root length and fresh and dry weight compared to the uninoculated control [141]. Additionally, inoculated plants showed a significant increase in leaf surface area and chlorophyll content compared to control. A high degree of colonization by the isolates was confirmed by the formation of dense microcolonies on the root surface according to a scanning electron microscopy analysis.

A strain of *Burkholderia arboris* isolated from soil samples dominated by *Pinus roxburghii* was shown to be effective when inoculated in mung bean seeds in a pot experiment [204]. The strain was characterized by the solubilization activities of K and Zn and the ability to produce siderophore.

A total of 25 non-rhizobial endophytic bacteria were isolated from the root nodules of cowpea, soybean, and mung bean [127]. *Staphylococcus*, *Bacillus*, *Streptomyces*, and *Acinetobacter* were genera present in mung bean nodules, which exhibit some characteristics related to plant growth promotion, such as Zn and P solubilization activities and auxin production. 

Under N-limited conditions, the shoot and root biomass of mung bean, cowpea, and soybean were significantly enhanced by the biofertilizer consisting of three strains (*B. japonicum*, *B. elkanii*, and *Streptomyces griseoflavus*) compared to the uninoculated control [205]. The application of biofertilizer also improved nodulation and nitrogen fixation in the three leguminous crops. Regarding grain yield, the authors recommend that the biofertilizer can be useful for both soybean and mung bean production.

Inoculation of mung beans with a consortium consisting of N_2_-fixing bacteria, P-solubilizing bacteria, and AMF promoted a 23.3% increase in grain yield compared to the uninoculated control [206]. In another study, inoculation with AMF improved the BNF efficiency of a *Bradyrhizobium* sp. strain, leading to improved nodulation, biomass, seed yield, and plant nutrition [151]. AMF inoculation combined with nutrient rich compost was also evaluated as a mung bean inoculant by Wahid et al. [207]. The treatment promoted an improvement in chlorophyll and carotenoid concentrations and shoot and root biomass and length compared to the control.

*Streptomyces thermocarboxydus* isolated from spores of *F. mosseae*, an AMF from the family Glomeraceae, showed the highest IAA and siderophore production and P-solubilization activity. The inoculation of mung beans with *S. thermocarboxydus* resulted in a significant increase in the length of the root and the total length (shoot and root), and the fresh weight as a consequence of IAA production, relative to the control [208].

Under salinity stress, inoculation of mung bean with PGPR and salt-tolerant bacteria resulted in an increase in growth, biomass, and physiology parameters, even at 2 and 10% salinity levels. Furthermore, the inoculated mung bean showed an increase in the uptake of N and P under saline conditions, mobilizing Na^+^ ions from root to shoot to reduce the toxicity presented by the ion [209].

Depending on the geographical region, nearly 40–100% of the losses in mung bean yield are due to various environmental stresses [210]. Drought stress is one of the most important factors that significantly affects agricultural land and reduces the production of various crops. Ahmed et al. [211] induced drought stress by adding a chemical PEG-6000 and evaluated the mitigation potential of a drought-tolerant bacterial consortium of *Enterobacter* sp. and *Leclercia adecarboxylata* recovered from rhizospheric soil, which produced significant amounts of plant growth-promoting bioactive compounds and colonized the roots of mung bean plants. According to the authors, these strains not only increase water and nutrient absorption, but also improve stomatal conductance, helping buffer the detrimental effects of drought.

The mixed inoculation of *F. mosseae* and *P. fluorescens* effectively alleviates the harmful effects of water stress. An enzyme assay suggested that the co-inoculation of *F. mosseae* and *P. fluorescens* was also effective in increasing the antioxidant defense system such as catalase, glutathione—and glutathione reductase [134]. These antioxidative enzymes are the most important components in the reactive oxygen species scavenging system [212,213].

#### 3.2.4. Pigeon Peas

In India, pigeon peas cultivated in acid soils, which received an inoculant consisting of *Rhizobium* strains and P-solubilizing bacteria, in addition to limestone application and a reduced dose of N (75%), showed an increase in nutrient absorption and the highest grain yield [214]. Economic analyzes were in line with the highest net return value and the best cost–benefit ratio. In sandy soil, tripartite inoculation of broad beans with *A. chroococcum*, *P. megaterium*, and *P. fluorescens* increases the rhizospheric abundance of N fixers and nitrifiers compared to control and mineral fertilizer application [136]. Furthermore, tripartite inoculation promoted the availability of rhizospheric N and P by 1.17 and 1.03, respectively, compared to the uninoculated control, while inoculation did not differ from the recommended dose of mineral fertilizer.

Broad bean inoculation with a consortium of *A. chroococcum*, *P. megaterium*, and *Pseudomonas* sp. was evaluated to reveal the synergistic mechanism among the three bacterial species [123]. The consortium promoted an increase in shoot and root length, and fresh and dry weigh compared to the inoculation of a single bacterium or two-membered consortium.

Sulfur deficiency negatively affects BNF in legumes, which depend on this element for the formation of sulfur amino acids and proteins. Strains characterized as sulfur oxidizing bacteria (SOB) convert elemental sulfur (S_0_) to sulfates (S^+6^), which occurs concomitantly with the reduction in the pH of the culture medium. *Stenotrophomonas* spp. strains stimulate the overexpression of sulfate transport genes in roots and sox genes in the rhizosphere of pigeon pea and act synergistically, increasing both absorption and translocation of sulfur from the roots to the aerial part of plants [215,216]. The inoculated plants showed an increase in N, P, and K uptake compared to the uninoculated control.

Broad bean inoculation with a consortium of P-solubilizing bacteria associated with *Rhizobium* increased the yield in a multilocus experiment during two consecutive years in India [217]. The grain yield was 50% higher when the bacterial inoculant was combined with the recommended dose of fertilizer and vermicompost (2 tons ha^−1^), compared to the control without fertilization, while the consortium inoculation increased grain yield by 17% compared to the uninoculated control.

Drought stress can be mitigated by inoculating pigeon pea seeds with PGPR (*B. aryabhattai* and *Bacillus* spp.) in a pot experiment containing soil at 50% and 25% of field capacity [218]. *B. aryabhattai* (IAA+) was more effective in promoting plant growth under stress than in soil at field capacity. Plants inoculated with *B. aryabhattai* showed a relative water content, 26.3% higher than the control. Different regulatory strategies were detected such as root biomass and relative water content (RWC) increase, and level reduction in osmolytes, proline, glycine-betaine, and antioxidants. Quantitative RT-PCR revealed that bacterial inoculation positively regulated gene expression in response to water shortage and negatively regulated proline gene expression.

Bacteria promote plant growth by reducing ethylene levels in plants growing under stress conditions due to the production of the enzyme ACC deaminase which converts ethylene into ammonia and α-ketobutyrate, reducing ethylene tissue levels [219]. In pigeon pea, the ACC deaminase activity of *Enterobacter indigenous* mitigates salinity stress under controlled and field conditions, in a loamy soil, which was corroborated by using cobalt chloride, an inhibitor of ethylene biosynthesis. 

## 4. Microbiomes Associated with Pulses

Plants live in close association with microbial communities that perform functions related to nutrition and protection against biotic and abiotic stresses [220]. Plant microbiomes are spread across plant tissues and organs; among the best known are the rhizosphere, nodule, root, and seed microbiomes. As interest grows in these populations and the benefits they bring to plants, the number of studies addressing this subject is increasing, aiming to understand the diversity and functionality of microbiomes, while at the same time seeking ways to use this knowledge to design new strategies to support plant development. 

### 4.1. Nodule Microbiome

N_2_-fixing bacteria are major components of nodule microbiomes, where they co-exist with other PGPRs. Analysis of nodule microbiomes may point to new N_2_-fixing strains, as well as other bacterial species that may act together to increase BNF activity. There is no consensus so far on the role of non-rhizobial bacteria present inside the nodules, although they have a high percentage of PGPR traits. As these communities are studied, the information can be used to develop promising biofertilizers composed of combinations of bacterial strains capable of providing a greater response potential than those with only a single N_2_-fixing strain. 

For example, data from metagenome analysis showed that *Bradyrhizobium* is predominant in mung bean and cowpea nodules regardless of the plant genotype or the history of the cultivation area [87,221]. The prevalence of this genus has previously been reported for three areas of mung bean production in Pakistan; however, *Ensifer*, a fast-growing rhizobium, represented a relative abundance of 99% in all rhizobial sequences in a sample collected from a desert region [222]. In Favero et al. [87], the genus *Pseudomonas* was the most abundant non-rhizobial bacteria (NRB) in mung bean observed in nodules from one cultivar, while Hakim et al. [222] showed *Acinetobacter* as the most abundant NRB, followed by *Microbacterium* and *Pseudomonas*. In cowpea, the bacterial groups *Microbacterium*, *Chitinophagales*, *Rhizobiaceae*, and *Acetobacteraceae* were present in the nodule microbiome, which was also influenced by the plant genotype. In contrast, the low and high rates of Cr-rich composted tannery sludge added to the soil did not affect the microbiome composition [221]. The presence of *Pseudomonas* in different nodule microbiomes is worth noting since species of this genus have several PGPR and biocontrol properties. When studying microbial communities in *Lotus burttii* nodules, Crosbie et al. [220] discovered that a representative of *Pseudomonas* was present in healthier plants and that it co-colonized nodules infected by an effective strain of *Mesorhizobium,* but not with an ineffective *Rhizobium* strain. Additionally, co-inoculation with the *Pseudomonas* strain decreased nodulation with the ineffective *Rhizobium* rather than the effective *Mesorhizobium*. 

In a recent study, the nodule and root microbiomes of soybeans and common beans were evaluated to compare the microbial colonization of plants inoculated with elite rhizobial strains and their co-inoculation with *A. brasilense* [43]. The nodules of the inoculated soybean showed a high abundance of the *Bradyrhizobium* strain, compared to the bean nodules. Co-inoculation slightly reduced the abundance of *Bradyrhizobium* in soybean nodules, while it did not change the percentage of bean nodules. For both crops, co-inoculation promoted a significant increase in rhizobial abundance in the roots. In beans, BNF is generally limited, and the authors suggested that it may be due to reduced rhizobial colonization. In broad beans, inoculation with Rlv resulted in an increase in seed N content and grain protein without an increase in BNF compared to the non-inoculated control [154]. Regardless of inoculation or not with a selected strain (*Rhizobium*, *Burkholderia*, or AMF) as a single inoculant or in co-inoculation, *Rhizobium* was the dominant genus in the nodule microbiome, suggesting that this genus was already present in the soil. NRB belonging to the genera *Pseudomonas*, *Devosia*, *Agrobacterium,* and *Rhodococcus* were also found. The inoculated treatment with a 20% reduction in the fertilizer dose did not decrease grain production, corresponding to an environmentally friendly alternative.

### 4.2. Root and Rhizosphere Microbiome

Several studies with root and rhizosphere microbiomes were performed with different pulses. These microbiomes are known to influence plant growth and productivity. Unlike the nodule microbiome, they can provide an evaluation of the impact caused by the production system and enable the selection of indicators related to the sustainability of agroecosystems. A new species-specific and highly polymorphic 16S-23S rRNA intergenic spacer barcode was designed to estimate the diversity of bradyrhizobial populations associated with the nodulated roots of cowpea and peanut plants in Senegal [86]. *B. vignae* is the dominant symbiont in the region. The use of barcoding also showed that the introduction of inoculants could modify the structure of bacterial populations. The difference found between the cowpea and peanut microbiomes confirms the influence of plant species on *Bradyrhizobium* genotypes.

For several plant species, including the common bean, Soldan et al. [223] propose that the rhizosphere microbiome may have been affected in modern cultivars compared to their wild ancestors. The effects of the characteristics of domesticated plants resulting from direct and indirect selection on the assembly of the host microbiome can lead to species loss, species gain, or species replacement, with the endophyte microbiota varying more among plant organs than among bean varieties [196,224]. Perez-Jaramillo et al. [225] showed that wild common bean relatives were enriched in bacterial taxa of the phylum *Bacteroidetes*, relative to modern relatives that were enriched in *Actinobacteria*. This change in composition was associated with plant genotypic traits as well as root phenotypic traits, showing a significant effect between common bean genotype and the associated microbial diversity.

Lv et al. [226] evaluated the structure and complexity of different root and nodule endophytes recruited during the life cycle. The *Rhizobium* genus was prevalent during all stages of development, being the highest relative abundance observed during flowering. The highest complexity was achieved at the seedling roots, which progressively decreased as the plant matured, whereas nodule complexity is also quite high during flowering. The relative abundance of *Pseudomonas* gradually increases during the life cycle, whereas that of *Bacteroides* decreases. From the dynamics established between endophytes, the authors consider that it is possible to propose the design of a synthetic community that can be used to promote activities to implement plant development, such as BNF and resistance to disease.

*R. irregularis*, used as an AMF inoculant in both phases of a rotation system composed of canola and pulses (pea or lentil), as the preceding crops, and corn and flax, as the main crops, resulted in the enrichment of the root microbiome with *Rhizobium* in the presence of pulses, which tends to be related to higher yields and the highest uptake of N and P in the main crops [227]. Microbiome studies can bring to light new information about complex systems involving several plant species that may be affected to a certain extent by patterns and dynamics established by microbial communities. The percentage of intercropping overyielding due to the soil microbial community was estimated [228]. In wheat and broad beans or corn and broad beans intercrops, the microbial legacy contributed with an increase of 28 to 51%. In intercropping, an increase in fungal diversity is generally observed compared to monocultures. Broad bean, in contrast to wheat and corn, benefits from cultivation with another crop. Wheat and corn intercropping did not show significant overyielding. The authors conclude that the dynamics established by both beneficial and pathogenic microorganisms are important in designing sustainable agricultural systems, usually based on the intercropping of legumes and cereals.

The rhizosphere microbiome may be a useful indicator of soil fertility and agroecosystem sustainability. During a long-term experiment maintained for at least eight years, the bacterial communities from the pea rhizosphere and the bulk soil were analyzed [229]. Rhizospheric bacterial diversity was higher compared to bulk soil, although different tillage and residue management treatments did not promote significant differences, suggesting that the key driver for alpha diversity levels should be the rhizosphere effect. The rhizosphere is characterized by a high abundance of *Rhizobium*, *Pseudomonas*, *Pantoea*, *Nitrobacter*, *Enterobacter*, and *Sphingomonas*. Conservation agriculture management predicts the maintenance of crop productivity, and, in this sense, the rhizospheric microbiome can be a highly sensitive indicator capable of anticipating changes in the sustainability status. When comparing the rhizosphere microbial communities of wheat (*Triticum aestivum*), rapeseed (*Brassica napus*), and pea, Wyszkowska et al. [230] demonstrated that wheat promoted the most beneficial effect on bacterial development and enzyme activities, indicated by the ecophysiological diversity index (EP, based on bacteria and actinobacteria counts) and the abundance of operational taxonomic units (OTU). The authors highlight that this index can be used as an indicator of soil fertility indicator. 

Rhizospheric soil samples from cowpea growing in a semiarid region of Pakistan were used as inoculant in controlled experiments subjected to saline stress conditions. Shoot length, plant biomass, and root nodules were higher when inoculated with two of the four soil samples collected [118]. The soil microbiome showed an overall pattern at the phylum level, being dominated by *Actinobacteria*, *Firmicutes*, and *Proteobacteria*, while the *Bradyrhizobiaceae* family is prevalent in the nodule microbiome of controlled experiments. The genera *Bosea*, *Afipia*, *Rhodopseudomonas*, and *Oligotropha* are also present. The authors suggested that the positive results obtained might be due to the presence of PGPR, which is a promising way to develop a cowpea inoculant with the aim of increasing tolerance under salinity stress.

In the rhizospheric community of cowpea grown after maize, a constant increase was observed in the fila *Proteobacteria*, *Armatimonadetes*, WPS-2, and OP11 between flowering and senescence [231]. There was a predominance of *Actinobacteria* and *Proteobacteria*. Network analysis showed that during the senescence phase, the rhizospheric community was the most complex, closely followed by the nodule microbiome during flowering. Keystone genera were identified, citing, among others, *Bacillus* for corn and cowpea and *Microlunatus* for cowpea related to P metabolism. These keystone genera are indicative of species that can contribute to the stabilization of microbial communities, which may have biotechnological applications.

Root architecture and exudation modified by plant domestication may have led to new microbial associations in the rhizosphere [232]. According to Medina-Paz et al. [233] the common bean recruits specific taxa from the surrounding soil within its native area and in a domestication area. Using 16S rRNA amplicon gene sequencing for paired evaluations of rhizosphere and endosphere communities at three ontogenetic stages in common beans, they found that the rhizosphere bacterial community was dominated by six phyla: *Proteobacteria* (41%), *Bacteroidetes* (14%), *Actinobacteria* (13%), *Gemmatimonadetes* (6%), *Chloroflexi* (4%), and *Acidobacteria* (3.5%). The dominant genera were Candidatus *Nitrososphaera* (11.5%), *Flavisolibacter* (3%), *Steroidobacter* (3%), *Kaistobacter* (2%), *Agrobacterium* (1%), and *Rubrobacter* (1%). In the endosphere, more than 99% of the sequences were annotated as *Proteobacteria* and most belonged to the genera *Agrobacterium* and *Rhizobium* (representing 30% each) followed by *Ochrobactrum* (less than 1%). Regarding the vegetative stage, the single out annotated as *Chryseobacterium* in the endosphere increased strongly and the abundance of rhizosphere OTUs annotated as *Rhizobium* and *Aeromonas* exhibited a 40-fold increase from the vegetative to the flowering stage.

### 4.3. Seed Microbiome

Seeds are colonized by microorganisms that can be transferred to the next generation, and contribute to their development, plant health, adaptation, and resistance to biotic and abiotic stresses and productivity. As we understand how seed microbiomes are organized, transferred, and preserved, we are better able to use this information in breeding programs to include the benefits of microbial communities in the development of new cultivars. In this sense, the seed microbiome is the first inoculum to colonize a germinating seed and may be responsible for initiating the microbiome assembly. When vertical transmission occurs directly to dormant seeds, it is possible to have a better estimate of the seed’s native population, reducing the influence of selection mechanisms that occur after the onset of germination [234]. 

The vertical transmission of seed bacterial communities across two generations of seven lentil genotypes cultivated in two different soil types has been investigated [234]. Although the seed microbiome differed significantly between soils, the genera *Cutibacterium*, *Methylobacterium*, *Sphingomonas*, *Streptococcus*, and *Tepidimonas* are transmitted and preserved in lentil genotypes. Despite the effect of soil, the occurrence of vertical transmission suggests the role of the seed microbiome in the adaptation and establishment of plant species.

Chartrel et al. [235] characterized pea seed microbiomes from different countries (France, Sweden, and Canada). While the bacterial core microbiome represents 31% of the relative abundance, the fungus core microbiome is around 82%, indicating that the variability is much higher in bacterial than in fungal communities. Additionally, the seed microbiome has a unique dominant microbial signature in each country. Crop, genotype, and field environmental conditions drive the seed microbiome, as demonstrated in wheat, canola, and lentil agricultural crops over two generations [236]. The interaction of genotype lines and generations explained the largest source of variation for both wheat and lentil microbial communities, corresponding to approximately 40 and 60% variance for bacteria and fungi, respectively. The core microbiome determined for each crop suggests that to some extent the host is responsible for selecting specific microorganisms, although the influence of environmental conditions must not be neglected.

The composition and structures of seedborne bacteria can vary between wild and domesticated species. For example, this variation has been observed for wild *C. judaicum* and domesticated *C. arietinum* from different geographic locations [237]. Diversity values were higher among domesticated genotypes compared to the wild genotypes. *Bacillus* spp. and *Sphingomonas* spp. dominated in the wild species, whereas *Burkholderia* dominated the domesticated species. The seed microbiome of wild species may provide clues for reconstructing the microbiomes of modern crops.

The effect of abiotic conditions (mild drought, half of baseline nutrition) on the structure of the bean seed microbiome for bacterial/archaeal communities was high compared to control, while for fungal communities the influence was limited [238]. No substantial changes in diversity were observed. The results shed light on the mechanisms capable of driving changes in the microbiome of plants under stress.

## 5. Conclusions

This review shows a broad research effort during the last five years aimed at selecting microsymbionts and plant growth-promoting microorganisms associated with nutritional aspects and tolerance to climate stress for pulses. The classical work for selecting rhizobial strains continues, and it has started to be integrated to the new field of microbiome studies, looking to study their interaction with the complex microbiota present in the rhizosphere. Still, microbiome studies in pulses are limited and deserve more attention due to their potential to contribute with new technologies, such as the design of synthetic microbial communities through the identification of keystone species responsible for structuring complex and generally more stable microbial networks. Other possible contributions are the use of microbiomes to mitigate climate change scenarios, and their integration into breeding programs.

Also, nodule endophytes have gained greater attention as a source of new microorganisms to improve the contribution of BNF. Approximately 65 PGPR taxa have been tested in the selected studies, the most common being *Pseudomonas* sp., *Bacillus* sp., *P. fluorescens*, *B. subtilis*, and *A. chroococcum.* Regarding arbuscular mycorrhizal fungi, a group that has received increasing attention, 13 taxa are listed, citing AMF sp. and *R. irregularis* as the most prevalent.

Strategies based on microorganisms for inoculation either as isolated strains or in multiple functional inoculants can increase the opportunities for implementing the use of biofertilizers in pulses. There is a consensus that, so far, there is not a clear picture capable of explaining the behavior of microbial communities associated with plants. However, establishing response patterns against biotic and abiotic factors will be essential to determine their functioning. These responses are urgent and necessary, especially if they are adaptable to the edaphoclimatic conditions of each region in the face of climate change scenarios, as to guarantee increased grain productivity and reducing the dependence on fertilizers, such as N, or increasing their use efficiency, such as in the case of P and K.

In recent years, a large amount of information has been generated in studies such as those discussed in this text. An attempt to see the various aspects related to the subject comprehensively can facilitate our understanding and contribute to future research focused on advancing the technological maturity level to promote the adoption of technologies capable of increasing grain productivity and protein supply capable, and at the same time, reflecting the economic, social, and environmental impact on pulse production systems worldwide.

The creation of a distribution program for pulses, seeds, and biofertilizers, especially in countries with limited food security, may be a critical and constructive approach capable of motivating fund managers, public or private.

## Figures and Tables

**Table 1:** Annual grain production of eight pulse legumes: beans, chickpeas, peas, cowpeas, mung beans, lentils, broad beans, and pigeon peas according to the FAOSTAT code. Data are averages for the period 2018 to 2020. Countries responsible for at least 80% of total production are shown.

FAO Code	Pulses	Grain Production (10^3^ Tons)	Number of Producing Countries	Main Producing Countries
176	Common beans (*Phaseolus vulgaris*); mung beans (*Vigna radiata*) *	27,038	103	India (20.9%), Brazil (10.9%), Myanmar (10.8%), China (4.9%), United Republic of Tanzania (4.4%), United States (4.4%), Mexico (3.9%), Kenya (2.9%), Uganda (2.7%), Argentina (2.1%), Ethiopia (2.0%), Burundi (1.8%), Rwanda (1.7%), Cameroon (1.5%), Mozambique (1.4%), Canada (1.4%), Angola (1.3%), Democratic People’s Republic of Korea (1.2%)
191	Chickpeas (*Cicer arietinum*)	15,402	46	India (70%), Turkey (4.1%), Myanmar (3.3%), Australia (3.2%), Russian Federation (3.1%)
187	Peas (*Pisum sativum*)	14,015	95	Canada (29.5%), Russia Federation (17.6%), China (10.5%), United States (6.5%), India (6.2%), France (4.6%), Ukraine (4.3%), Ethiopia (2.7%)
195	Cowpeas (*Vigna unguiculata*)	8578	35	Nigeria (41.5%), Niger (28.8%), Burkina Faso (7.8%), Kenya (2.6%)
201	Lentils (*Lens culinaris*)	6297	42	Canada (39.4%), India (21.3%), Australia (7.6%), Turkey (5.7%), United States (5.1%), Nepal (4.0%)
181	Broad beans (*Vicia faba*)	5531	64	China (31.8%), Ethiopia (18.8%), United Kingdom of Great Britain and Northern Ireland (9.1%), Australia (5.8%), Germany (3.3%), Lithuania (3.0%), Sudan (3%), France (2.8%), Italy (2.3%)
197	Pigeon peas (*Cajanus cajan*)	4917	24	India (77.9%, Malawi (8.6%), Myanmar (7.1%)

* Classification of pulses according to FAO: common bean and mung bean production share the same FAO code and are accounted together with lima bean (*Phaseolus lunatus*), scarlet runner bean (*Phaseolus coccineus*), tepary bean (*Phaseolus acutifolius*), adzuki bean (*Vigna angularis*), mungo bean (*Vigna mungo*), rice bean (*Vigna umbellate*), and moth bean (*Vigna aconitifolia*).

**Table 2:** Main microbial taxa described in interactions with pulses selected for their PGPR characteristics.

		Pulses (References)
Bacteria	*Pseudomonas*	chickpea [111,112,113]; pea [114,115]; lentil [116]; common bean [117]; cowpea [118,119,120]; mung bean [121,122]; pigeon pea [123]
	*Bacillus*	chickpea [111]; pea [114,124]; lentil [125]; cowpea [119]; common bean [43,117,126]; mung bean [122,127]
	*Pseudomonas fluorescens*	chickpea [128,129]; broad bean [130]; lentil [63,131]; common bean [132,133]; mung bean [134,135]; pigeon pea [136]
	*Bacillus subtilis*	chickpea [137]; pea [115,138]; broad bean [139]; common bean [132,140]; mung bean [135,141].
	*Azotobacter chroococcum*	chickpea [137]; broad bean [142]; common bean [143]; pigeon pea [123,136]
AMF	AMF	chickpea [144]; pea [145,146,147]; broad bean [148,149]; cowpea [78,150]; mung bean [151]
	*Rhizophagus irregularis*	Chickpea [112]; pea [152]; broad bean [153,154]

## Data Availability

No new data were created or analyzed in this review.
